# Characterization and analysis of guideline implementation tools (GItools) reveals opportunities for improving health service planning, delivery and quality improvement

**DOI:** 10.1186/1472-6963-14-S2-O8

**Published:** 2014-07-07

**Authors:** Anna R Gagliardi

**Affiliations:** 1 Toronto General Research Institute, University Health Network, Toronto, Canada

## Background

Guidelines inform health care planning, delivery and quality improvement but are not consistently implemented [1-3]. Research shows that guidelines were more likely to be used when accompanied by guideline implementation tools (GItools), but few guidelines offered GItools [4-6]. Interviews with guideline developers and analysis of guideline instructional manuals revealed a need for information to support GItool development [7-9]. First it is necessary to characterize GItools. The purpose of this research was to generate a framework of desirable GItool features, and use the framework to describe a sample of GItools.

## Materials and methods

Items representing desirable GItool features were first generated by a cross-sectional survey of the international guideline community [10,11]. Then items were confirmed and refined by a panel of guideline developers, implementers and researchers in a two-round Delphi survey [12-14]. The resulting GItool framework was applied to describe a sample of GItools of various types, accompanying guidelines identified in the National Guideline Clearinghouse on various clinical topics produced within five years by organizations having developed at least ten guidelines.

## Results

The cross-sectional survey was completed by 96 respondents from Australia, Canada, United Kingdom, United States, The Netherlands, and several other countries. Nine items were rated by most as desirable but difficult to achieve given limited resources and a perceived imperative to make GItools accessible even if not rigorously developed or evaluated. Thirty-one panelists from ten countries including Australia, Canada, Germany, New Zealand, Peru, Saudi Arabia, Spain, United Kingdom, and the United States took part in a two-round Delphi survey. Twelve items achieved consensus as desirable GItool features. A total of 13 GItools were identified among a sample of 149 guidelines (8.7%). Most GItools named target users (92.3%) and described development methods (84.6%). Fewer possessed other features considered desirable such as instructions for use (61.5%), sources of content (61.5%), target users were involved in development (53.8%), underlying evidence identified (23.1%), evaluation described (7.7%), or pilot-tested with target users (0.0%).

## Conclusions

Further work is needed to validate the framework with guideline users, and share the GItool framework with guideline developers. It can serve as the basis for evaluating and adapting existing GItools, or developing new GItools. Inclusion of higher quality GItools with more guidelines may support implementation and use of guidelines by target users, ultimately leading to improved care delivery and associated outcomes.
